# Robust multiferroic in interfacial modulation synthesized wafer-scale one-unit-cell of chromium sulfide

**DOI:** 10.1038/s41467-024-44929-5

**Published:** 2024-01-24

**Authors:** Luying Song, Ying Zhao, Bingqian Xu, Ruofan Du, Hui Li, Wang Feng, Junbo Yang, Xiaohui Li, Zijia Liu, Xia Wen, Yanan Peng, Yuzhu Wang, Hang Sun, Ling Huang, Yulin Jiang, Yao Cai, Xue Jiang, Jianping Shi, Jun He

**Affiliations:** 1 The Institute for Advanced Studies, Wuhan University, Wuhan, 430072 China; 2 Key Laboratory of Materials Modification by Laser, Ion and Electron Beams (Ministry of Education), Dalian University of Technology, Dalian, 116024 China; 3 Key Laboratory of Artificial Micro- and Nano-Structures of Ministry of Education, School of Physics and Technology, Wuhan University, Wuhan, 430072 China; 4 The Institute of Technological Sciences, Wuhan University, 430072 Wuhan, China

**Keywords:** Ferroelectrics and multiferroics, Ferroelectrics and multiferroics, Synthesis and processing

## Abstract

Multiferroic materials offer a promising avenue for manipulating digital information by leveraging the cross-coupling between ferroelectric and ferromagnetic orders. Despite the ferroelectricity has been uncovered by ion displacement or interlayer-sliding, one-unit-cell of multiferroic materials design and wafer-scale synthesis have yet to be realized. Here we develope an interface modulated strategy to grow 1-inch one-unit-cell of non-layered chromium sulfide with unidirectional orientation on industry-compatible *c*-plane sapphire. The interfacial interaction between chromium sulfide and substrate induces the intralayer-sliding of self-intercalated chromium atoms and breaks the space reversal symmetry. As a result, robust room-temperature ferroelectricity (retaining more than one month) emerges in one-unit-cell of chromium sulfide with ultrahigh remanent polarization. Besides, long-range ferromagnetic order is discovered with the Curie temperature approaching 200 K, almost two times higher than that of bulk counterpart. In parallel, the magnetoelectric coupling is certified and which makes 1-inch one-unit-cell of chromium sulfide the largest and thinnest multiferroics.

## Abstract (web summary)

2D multiferroic materials have garnered broad interests due to their magnetoelectric properties and multifunctional applications. Here, the authors discover a multiferroic feature in interfacial modulation synthesized wafer-scale one-unit-cell Cr_2_S_3_.

## Introduction

Multiferroic materials are well known for their potential applications in non-volatile memories and sensors, which have been extended to the fields of photovoltaics for efficient renewable energy harvesting and synaptic devices for powerful neuromorphic computing^1–3^. Nevertheless, the existing three-dimensional (3D) multiferroic materials fall short of meeting the industry criteria for practical applications in information storage because of the size limit, interfacial effect, polarization origin, and reversal mechanism^2,4,5^. Two-dimensional (2D) materials have revealed an unprecedented potential to consistently drive advanced device performances due to their unique physical and electronic properties^6–9^. Particularly, 2D ferroelectricity has been uncovered and the spontaneous polarization generally originates from the non-centrosymmetric structure^10–15^. In addition, the weak interlayer interaction in 2D layered materials enables the layer-sliding, which breaks the centrosymmetry and leads to the emergence of polarization^16–23^. Even so, experimental exploration of 2D multiferroic materials still remains a daunting challenge especially at the atomically thin thickness because of the influence of thermal fluctuation and depolarizing field^24^. Furthermore, the relatively small remanent polarization in 2D sliding ferroelectrics limits their practical applications. In this regard, searching for 2D intrinsic multiferroic materials has become the central task for constructing the next-generation information storage devices.

Chromium-based chalcogenides (Cr_*m*_X_*n*_, where X = S, Se, and Te) possess tunable structural phases and magnetic orders by means of the stoichiometric variation^25–30^. The alternating stacks of Cr-deficient and Cr-full layers, as well as the self-intercalation of Cr atoms determine such interesting magnetic properties. Interestingly, the intralayer-sliding of self-intercalated Cr atoms may break the space reversal symmetry and induces the spontaneous polarization. Assuming this to be the case, one-unit-cell of Cr_*m*_X_*n*_ should be one of the thinnest multiferroic materials. However, the experimental exploration is yet to be realized in view that the strong chemical bond between Cr and X restricts the intralayer-sliding. On the other hand, to meet the industry criteria for practical applications in information storage, the batch synthesis of wafer-scale 2D multiferroic single crystals is a fundamental issue. Despite considerable efforts have been devoted^31–34^, the wafer-scale growth of one-unit-cell of non-layered Cr_*m*_X_*n*_ still remains challenging because the inherent 3D chemical bond hinders the 2D anisotropic growth.

Here we develop an interface-modulated chemical vapor deposition (CVD) method to synthesize 1-inch one-unit-cell of non-layered chromium sulfide (Cr_2_S_3_) with unidirectional orientation on *c*-plane sapphire. The relatively strong interfacial interaction is demonstrated between Cr_2_S_3_ and substrate, which determines the unidirectional growth of Cr_2_S_3_ and intralayer-sliding of self-intercalated Cr atoms. As expected, the space reversal symmetry is broken and the room-temperature ferroelectricity emerges in one-unit-cell of Cr_2_S_3_ featured with ultrahigh stability (retaining more than one month). Meanwhile, the long-range ferromagnetic order is uncovered with the Curie temperature approaching 200 K, almost two times higher than that of bulk counterpart. Combining density functional theory (DFT) calculations and low-temperature quantum transport/piezoresponse force microscopy (PFM) measurements, the internal mechanism of multiferroic is clarified unambiguously. This work provides an innovative strategy for bridging synthesis and multiferroic investigation of wafer-scale one-unit-cell of non-layered Cr_2_S_3_, which enables the construction of multi-terminal spintronic chips and magnetoelectric devices.

## Results

### Synthesis of 1-inch length one-unit-cell of non-layered Cr_2_S_3_

**Fig. 1:**
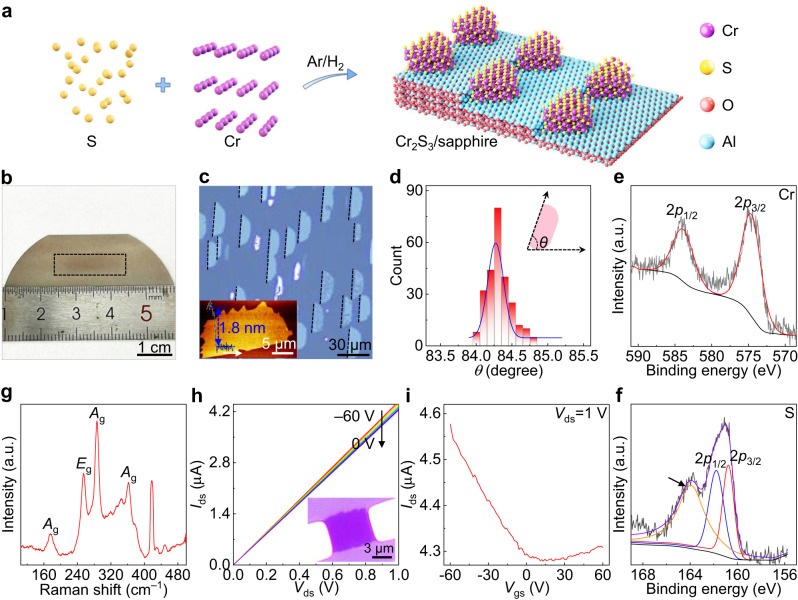
Orientation-controlled synthesize 1-inch length one-unit-cell of non-layered Cr_2_S_3_ on *c*-plane sapphire. **a** Schematic diagram of unidirectionally aligned growth of one-unit-cell of Cr_2_S_3_. **b** Photography of CVD-synthesized 1-inch length Cr_2_S_3_ on *c*-plane sapphire. **c** OM image of Cr_2_S_3_ on *c*-plane sapphire, showing the unidirectionally aligned feature. Inset: AFM image and corresponding height profile of a single Cr_2_S_3_ nanosheet, revealing its one-unit-cell nature. **d** Statistical analysis of domain orientations of Cr_2_S_3_ on *c*-plane sapphire. **e**, **f** XPS spectra of as-grown samples, showing the formation of Cr_2_S_3_ and Al-S bonds. **g** Raman spectrum of as-grown sample. **h**, **i** Output and transfer characteristic curves of a Cr_2_S_3_ back-gated FET. Inset: OM image of the device.

The industry-compatible *c*-plane sapphire is selected as the substrate because of its atomically flat surface and low surface diffusion barrier of reactants, which contribute to the evolution of atomically thin Cr_2_S_3_. Corresponding rocking curve is shown in Supplementary Fig. 1, where the miscut angles towards *A* and *M* axes are measured to be 0° and 0.2°, respectively. Notably, the parallel steps are readily formed on sapphire surfaces at relatively low temperature (980 °C) without any pre-annealing process (Fig. 1a and Supplementary Fig. 2), which is fundamentally important for the nucleation and growth of unidirectionally aligned Cr_2_S_3_^32–35^. Figure 1b reveals the digital photography of 1-inch length Cr_2_S_3_ on *c*-plane sapphire, with the optical microscopy (OM) images shown in Fig. 1c and Supplementary Figs. 3, 4, respectively. The half circular Cr_2_S_3_ nanosheets are observed on *c*-plane sapphire possibly due to the high energy barrier of passing over the step edge of sapphire. The obtained Cr_2_S_3_ nanosheets demonstrate nearly 100% unidirectional alignment feature on *c*-plane sapphire, different from the other 2D materials with random orientations^36^. This is further corroborated by the domain orientation distribution in Fig. 1d. In addition, the carrier gas flow direction reveals negligible influence on the domain orientation of Cr_2_S_3_ (Supplementary Fig. 5), indicating that the Cr_2_S_3_-sapphire interaction and parallel steps dominate the unidirectional aligned growth. Meanwhile, the largest Cr_2_S_3_ nanosheet is obtained with the domain size of 108 μm, as shown in Supplementary Fig. 6. In contrast, small domain-sized triangular or irregular Cr_2_S_3_ nanosheets with random orientations are evolved on Au foil, mica, and highly oriented pyrolytic graphite (HOPG) under the same condition, suggestive of the advantage of sapphire for growing unidirectionally aligned Cr_2_S_3_ (Supplementary Fig. 7). Atomic force microscopy (AFM) image and corresponding height profile analysis in Fig. 1c show that the thickness of Cr_2_S_3_ is 1.8 nm, corresponding to one-unit-cell nature.

To determine the chemical state and elemental composition, X-ray photoelectron spectroscopy (XPS) measurements were performed on as-grown samples, with the results shown in Fig. 1e, f. The binding energies at 574.8 and 584.1 eV are attributed to Cr^3+^, while the peaks of 160.8 and 161.8 eV are assigned to S^2–^, consistent with the XPS results of CVD-synthesized Cr_2_S_3_ on mica^28^. Nevertheless, a new characteristic peak (164.0 eV) is observed in the S 2*p* XPS spectrum (indicated by the black arrow in Fig. 1f), which derives from the Al-S bonds (Supplementary Fig. 8). Such a result manifests the strong interfacial interaction between Cr_2_S_3_ and sapphire, which determines the unidirectionally aligned growth of Cr_2_S_3_^37^. To further confirm the strong interfacial interaction determining the domain orientation of Cr_2_S_3_, Cr_2_S_3_/WS_2_ vertical heterostructures are synthesized on *c*-plane sapphire and the obtained Cr_2_S_3_ nanosheets possess random orientations (Supplementary Fig. 9). Cross-identification by Raman spectroscopy confirm the formation of Cr_2_S_3_ on *c*-plane sapphire, as shown in Fig. 1g and Supplementary Fig. 10. Notably, the characteristic peaks of Al_2_S_3_ are observed in the Raman spectrum of as-grown one-unit-cell of Cr_2_S_3_ on sapphire, further verifying the formation of Al-S bonds. To clarify the electronic properties of Cr_2_S_3_, a back-gated field-effect transistor (FET) was fabricated using Cr/Au as electrodes. The semiconducting feature with p-type is convinced by the output and transfer characteristic curves in Fig. 1h, i, as well as the temperature-dependent resistance in Supplementary Fig. 11. Briefly, 1-inch length one-unit-cell of non-layered Cr_2_S_3_ semiconductors with unidirectional orientation has been synthesized on industry-compatible *c*-plane sapphire, which provides a strategy for growing wafer-scale single crystals and a platform for constructing high-performance in-memory devices.

### Growth mechanism of unidirectionally aligned Cr_2_S_3_

**Fig. 2:**
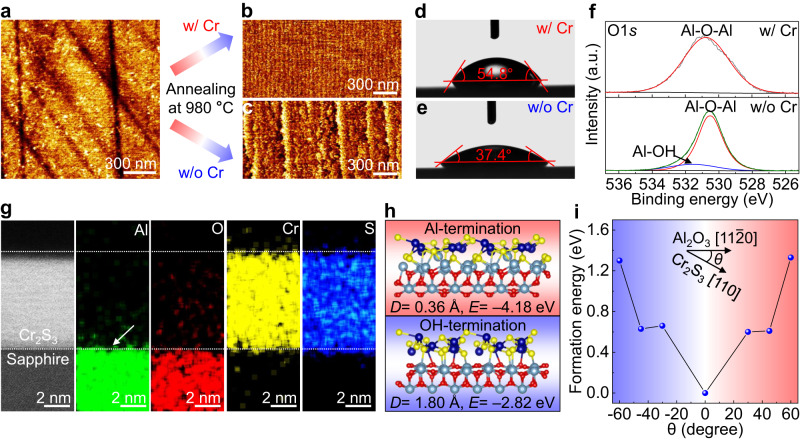
Growth mechanism of unidirectional aligned Cr_2_S_3_ on *c*-plane sapphire. **a** AFM image of an initial *c*-plane sapphire. **b**, **c** AFM images of *c*-plane sapphire after annealing at 980 °C with and without the presence of Cr powders, respectively. **d**, **e** Contact angles of distilled water droplet on these two distinct sapphire surfaces. **f** O 1*s* XPS spectra captured on these two distinct sapphire surfaces. **g** Cross-sectional STEM image and corresponding element distributions of as-grown Cr_2_S_3_ on *c*-plane sapphire. **h** Schematic diagrams of Cr_2_S_3_ on these two distinct sapphire surfaces. **i** Formation energy of Cr_2_S_3_ island with different rotation angles on *c*-plane sapphire, where *θ* is defined as the angle between [110] direction of Cr_2_S_3_ and [11–20] direction of *c*-plane sapphire.

The interfacial modulation method has been proposed to improve the quality of target materials (e.g. HgCdTe)^38,39^, which provides a direction to understand the growth mechanism of well-aligned Cr_2_S_3_ on *c*-plane sapphire. The AFM image of an initial *c*-plane sapphire is shown in Fig. 2a, where no steps is observed. Interestingly, the continuous and parallel steps are evolved on sapphire surfaces after annealing at 980 °C with the presence of Cr powders (Fig. 2b). By contrast, the discontinuous and distorted steps are formed under the same annealing condition without the presence of Cr powders (Fig. 2c). This result suggests that the introduction of Cr promotes the formation of parallel steps on sapphire surfaces, which play a crucial role for synthesizing unidirectionally aligned Cr_2_S_3_. To further expound the surface chemical property discrepancy, the water contact angle measurements were then performed on these two distinct sapphire surfaces. A larger contact angle is obtained on the sapphire surface (54.8°) after annealing with the presence of Cr powders than that on the counterpart (37.4°) after annealing without the presence of Cr powders (Fig. 2d, e and Supplementary Fig. 12), possibly due to the variation of sapphire surface-terminated structure.

In addition, the chemical state of sapphire surface was also detected by XPS, with the results shown in Fig. 2f. Two characteristic peaks corresponding to Al-OH (531.5 eV) and Al-O-Al (530.5 eV) are observed in the O 1*s* spectrum, which is obtained on the sapphire surface after annealing without the presence of Cr powders. This is totally different from the sapphire surface after annealing with the presence of Cr powders, where only one characteristic peak (530.5 eV) is observed. Such results indicate that the sapphire surface structure is changed from OH- to Al-termination after annealing with the presence of Cr powders, and the similar phenomenon is also demonstrated for high-temperature pre-annealing process^40^. Meanwhile, the formation of Al-S bonds further verifies the Al-terminated surface structure of sapphire. The cross-sectional scanning transmission electron microscopy (STEM) and corresponding energy-dispersive spectroscopy (EDS) characterization results reveal that Al elements are assembled at the interface between Cr_2_S_3_ and sapphire, as shown in Fig. 2g. The atomic-resolution cross-sectional STEM image in Supplementary Fig. 13 further verifies the formation of Al-terminated structure. DFT calculations were then carried out to clarify the growth mechanism of Cr_2_S_3_ on *c*-plane sapphire (Fig. 2h, i and Supplementary Fig. 14). A smaller Cr_2_S_3_-sapphire distance (*D*) is achieved on the Al-terminated surface (0.36 Å) than that on the OH-terminated surface (1.80 Å). Meanwhile, the adsorption energy (*E*) of Cr_2_S_3_ on Al-terminated surface is calculated to be –4.18 eV, much higher than that on OH-terminated surface (–2.82 eV). The small Cr_2_S_3_-sapphire distance and high adsorption energy manifest the strong interfacial interaction between Cr_2_S_3_ and sapphire, which contributes to the unidirectionally aligned growth of Cr_2_S_3_. Besides, the formation energies of Cr_2_S_3_ island with different rotation angles on *c*-plane sapphire were also calculated (Fig. 2i), demonstrating the crucial role of parallel steps on *c*-plane sapphire surfaces for the epitaxial growth of one-unit-cell thick Cr_2_S_3_ with unidirectional orientation. Particularly, unidirectionally aligned Cr_2_Se_3_ nanosheets have also been synthesized on *c*-plane sapphire using the same approach, indicating the universality of this interface-modulated growth strategy (Supplementary Fig. 15).

### Multiscale determining unidirectional alignment and seamless stitching of Cr_2_S_3_

**Fig. 3:**
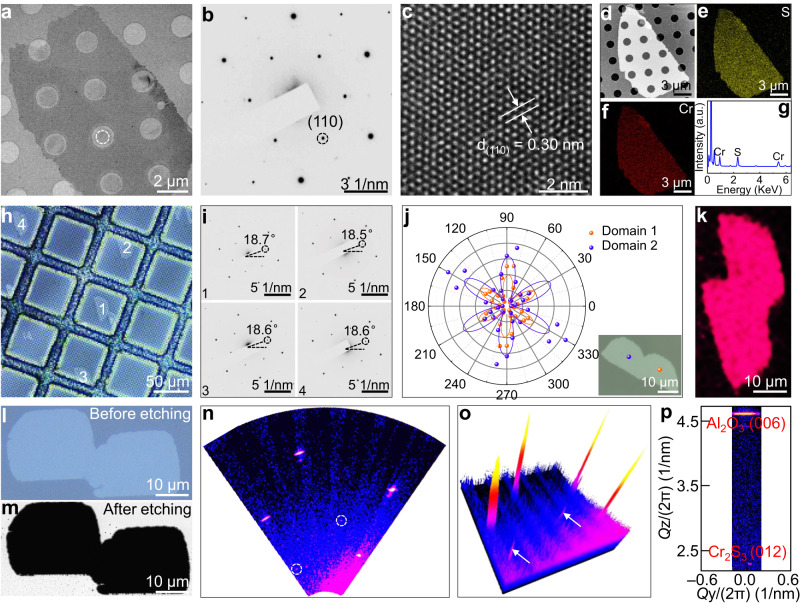
Unidirectional alignment and seamless stitching of Cr_2_S_3_. **a** Low-magnification TEM image of a transferred Cr_2_S_3_ nanosheet. **b** Corresponding SAED pattern captured from the circled region in (**a**), indicating its single-crystal feature. **c** Atomic-resolution TEM image of a transferred Cr_2_S_3_ nanosheet, showing its high crystalline quality. **d** Low-magnification STEM image of a transferred Cr_2_S_3_ nanosheet. **e**, **f** Corresponding EDS mapping images of Cr and S, revealing the uniform element distributions. **g** Quantified elemental analysis of Cr_2_S_3_. **h** OM image of transferred Cr_2_S_3_ nanosheets. **i** Corresponding SAED patterns captured from different Cr_2_S_3_ nanosheets, showing nearly the identical orientations. **j**, **k** Polarized SHG spectra and mapping image measured on two merged Cr_2_S_3_ domains with unidirectional alignment. Inset: OM image of such two merged domains. **l**, **m** OM and scanning electron microscopy images of two merged Cr_2_S_3_ domains with unidirectional alignment before and after Ar/O_2_ etching. **n**–**p** RSM results of as-grown Cr_2_S_3_ on sapphire, highlighting the unidirectional alignment of Cr_2_S_3_ at millimeter scale.

To explore the atomic structure and unidirectional alignment of CVD-synthesized one-unit-cell of Cr_2_S_3_, multiscale characterizations were thus performed on as-grown and transferred samples. Figure 3a shows the low-magnification TEM image of a transferred Cr_2_S_3_ nanosheet, the well-defined morphology and uniform color contrast indicate its high crystalline quality and thickness uniformity. The corresponding selected-area electron diffusion (SAED) pattern reveals only one set of spots, suggestive of its single-crystal feature (Fig. 3b and Supplementary Fig. 16). As exhibited by the atomic-resolution TEM image in Fig. 3c, a perfect honeycomb lattice is observed to show almost no visible defect, indicating the high crystalline quality. The (110) lattice plane spacing is calculated to be 0.30 nm, consistent with CVD-synthesized Cr_2_S_3_ nanosheets on mica^28^. To further identify the element constitutions and their distributions, EDS characterizations were then performed, with the results shown in Fig. 3d–g and Supplementary Fig. 17. The uniform color contrast within the nanosheet manifests the high crystalline quality of CVD-derived Cr_2_S_3_. The atomic ratio of Cr to S revealed by quantified elemental analysis is calculated to be 38.57:61.43 ≈ 2:3, demonstrating the perfect stoichiometric ratio of Cr_2_S_3_.

A series of SAED patterns captured from different nanosheets reveal nearly the identical orientations, confirming the unidirectional alignment of Cr_2_S_3_ (Fig. 3h, i and Supplementary Fig. 18). To realize the wafer-scale synthesis of single crystal, the seamless stitching of unidirectional aligned nanosheets is crucial^32,35,41^. The polarized second-harmonic generation (SHG) spectra captured from two merged Cr_2_S_3_ domains reveal nearly the identical patterns, indicating the same orientations (Fig. 3j). Meanwhile, the corresponding SHG mapping reveals no obvious intensity drop across the grain boundary, confirming the seamless stitching of such two Cr_2_S_3_ domains (Fig. 3k). In contrast, the SHG spectra measured on two misoriented Cr_2_S_3_ domains exhibit a twist angle and a sharp boundary (Supplementary Fig. 19a, b). To further verify the seamless stitching of unidirectional aligned Cr_2_S_3_, the Ar/O_2_ etching experiments were then performed, and the grain boundary was obviously observed for the two merged Cr_2_S_3_ domains with random orientations (Supplementary Fig. 19c, d). Nevertheless, for the unidirectionally aligned Cr_2_S_3_, almost no contrast variation is presented, indicating their seamless stitching, as shown in Fig. 3l, m. Besides, the reciprocal space mapping (RSM) characterizations were also conducted on as-grown samples to convince the unidirectionally aligned feature of Cr_2_S_3_ at millimeter scale (Fig. 3n, o). The single diffraction pattern and the same direction between Cr_2_S_3_ (012) and Al_2_O_3_ (006) suggest the epitaxial growth mode of unidirectionally aligned Cr_2_S_3_ on *c*-plane sapphire (Fig. 3p). In brief, the achievement of 1-inch length one-unit-cell of Cr_2_S_3_ with unidirectional orientation and their seamless stitching provide the cornerstone for synthesizing wafer-scale 2D single crystals.

### Room-temperature ferroelectricity in one-unit-cell of Cr_2_S_3_

**Fig. 4:**
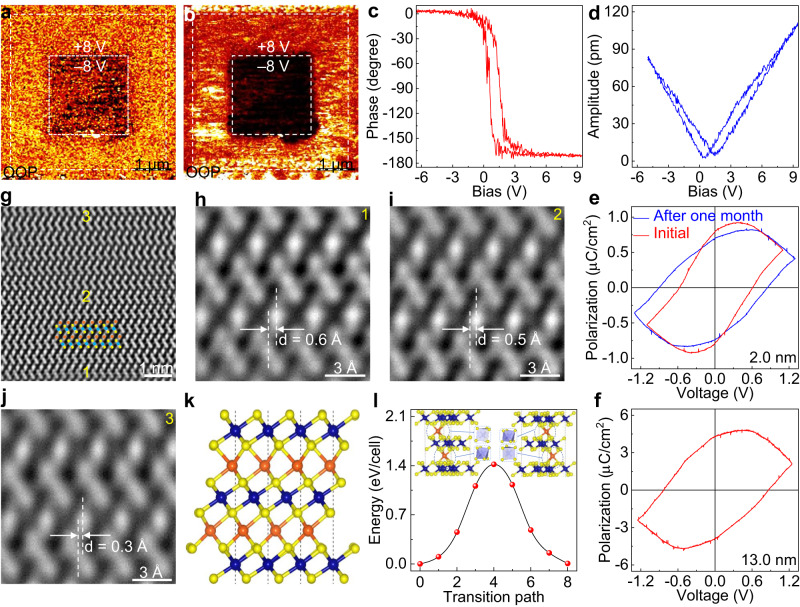
Room-temperature ferroelectricity in CVD-synthesized one-unit-cell of non-layered Cr_2_S_3_. **a**, **b** PFM phase and amplitude images of one-unit-cell of Cr_2_S_3_ after poling with ±8 V, indicating its stable polarization state. **c**, **d** Corresponding PFM phase and amplitude hysteresis loops of one-unit-cell of Cr_2_S_3_. **e**, **f** Macroscopic polarization hysteresis loops of Cr_2_S_3_ with the thicknesses of 2.0 nm and 13.0 nm, respectively, showing its robust and intrinsic room-temperature ferroelectricity. **g**–**j** Cross-sectional STEM images of as-grown Cr_2_S_3_ on *c*-plane sapphire, displaying the intralayer-sliding of self-intercalated Cr atoms. **k** Atomic structure of one-unit-cell of Cr_2_S_3_ with intralayer-sliding of self-intercalated Cr atoms. The blue, yellow, and orange spheres represent the Cr, S atoms in CrS_2_ layers and the self-intercalated Cr atoms, respectively. **l** Energy evolution between the two opposite polarization states of one-unit-cell of Cr_2_S_3_. The energy decreasing from the centrosymmetric to non-centrosymmetric structure indicates a continuous and spontaneous phase transition between these two phases.

The symmetry of Cr_2_S_3_ (with the thickness changing from 1.9 to 12.3 nm) was investigated by nonlinear optical SHG measurements (Supplementary Fig. 20). A prominent characteristic peak is observed at 532 nm (corresponding to the half of excitation wavelength) for all the testing samples, indicating the non-centrosymmetric structure of Cr_2_S_3_. The unusual space reversal symmetry broken possibly induces the emergence of ferroelectricity and then PFM measurements are performed on transferred samples onto Au/Si, with the schematic diagram shown in Supplementary Fig. 21. Notably, in view of the strong interfacial interaction between Cr_2_S_3_ and sapphire surface, the polypropylene carbonate (PPC) with high viscosity is selected as the supporting layer and the detailed etching-free transfer process is described in Supplementary Fig. 22. The PFM phase image after a box-in-box writing with a tip bias of positive and negative voltages (8 V) shows a well-defined region of phase contrast, corresponding to the remanent polarization (Fig. 4a). This result indicates that the polarization state can be rewritten, highlighting the switchable polarization feature of ultrathin Cr_2_S_3_ especially down to one-unit-cell thickness, which is also confirmed by the amplitude image in Fig. 4b. In addition, the butterfly loops of amplitude signal and the distinct 180° switching of phase corroborate the ferroelectric polarization in one-unit-cell of Cr_2_S_3_ (Fig. 4c, d). Notably, the interfacial charge and internal electric field induced by the distinct electrodes (PFM tip and Au) result in the asymmetry of PFM phase and amplitude hysteresis loops. To the best of our knowledge, it is the first report about the ferroelectricity of Cr_*m*_X_*n*_. All the testing Cr_2_S_3_ nanosheets with different thicknesses show the room-temperature ferroelectricity, nevertheless, the polarization signal gradually weakens with increasing the thickness (Supplementary Fig. 23). As the thickness reaches to 73.9 nm, no hysteresis loop or butterfly-like amplitude curve is observed (Supplementary Fig. 24).

To further verify the ferroelectricity in CVD-derived 2D Cr_2_S_3_, the macroscopic ferroelectric hysteresis loop was measured, and the remanent polarization value of one-unit-cell of Cr_2_S_3_ was calculated to be 0.80 μC/cm^2^ (Fig. 4e). Particularly, the ferroelectricity is still maintained even after one month and the remanent polarization shows negligible variation, as revealed in Fig. 4e, indicating the robust room-temperature ferroelectricity in 2D Cr_2_S_3_. The remanent polarization value as large as 4.30 μC/cm^2^ is observed for the Cr_2_S_3_ nanosheet with the thickness of 13.0 nm (Fig. 4f), which is higher than other 2D ferroelectric materials (Table 1). Besides of the interfacial interaction, the ionic polar displacement and interlayer charge transfer possibly contribute to enhance the polarization of thick Cr_2_S_3_, and related theoretical explorations are expected to be made in the future. Furthermore, the cumulative effect of self-intercalated Cr atoms and CrS_2_ layers sliding, as well as the weakened depolarization field should also result in the polarization elevation, as have been demonstrated in other ferroelectric materials^42,43^. In addition, the macroscopic ferroelectric hysteresis loop measurement of bulk Cr_2_S_3_ is performed, with the result shown in Supplementary Fig. 25. The counterclockwise hysteresis loops of transfer characteristic curves at different sweep rates and drain-source voltages are obtained in the vertical FET, and the large hysteresis window (40 V) further verifies the ferroelectric polarization in Cr_2_S_3_ (Supplementary Fig. 26).

**Table 1:** The remanent polarization and stability comparison for Cr_2_S_3_ with other 2D ferroelectric materials

Material	Thickness	Remanent polarization	Stability	Method	References
1 T’-ReS_2_	Multilayer	0.68 pC/m	/	DFT	^54^
Graphene/BN	Bilayer	0.18 μC/cm^2^	/	Device	^17^
BN	Bilayer	0.68 μC/cm^2^	/	Device	^18^
VS_2_	Bilayer	0.202 μC/cm^2^	/	DFT	^55^
MoS_2_/WS_2_	Bilayer	1.45 pC/m	/	Device	^20^
WSe_2_	Trilayer	0.53 pC/m	/	KPFM	^16^
Cr_2_S_3_	2.0 nm	0.80 μC/cm^2^	One month	Device	This work
	13.0 nm	4.30 μC/cm^2^			

To uncover the origin of ferroelectricity in 2D Cr_2_S_3_, the atomic-resolution cross-sectional STEM measurements were performed, with the results shown in Fig. 4g–j. Interestingly, unexpected intralayer-sliding of self-intercalated Cr atoms is observed, especially at the Cr_2_S_3_/sapphire interface (Fig. 4h). Away from the interface, the intralayer-sliding value of self-intercalated Cr atoms is reduced accordingly (from 0.6 to 0.3 Å), which should result in the attenuation of ferroelectricity. The strong interfacial interaction between Cr_2_S_3_ and sapphire surface induces such an unusual intralayer-sliding of self-intercalated Cr atoms. The similar phenomena have also been demonstrated in monolayer GaSe^44^ and γ-InSe^45^. Besides, DFT calculations were carried out to further understand the internal mechanism of ferroelectricity in 2D Cr_2_S_3_ (Fig. 4k, l). Comparing with the pristine AAA stacking of Cr_2_S_3_ (Supplementary Fig. 27a), the strong interfacial interaction between Cr_2_S_3_ and sapphire surface induces the self-intercalated Cr atoms sliding and a new ABA stacking order is built. The intercalated Cr atom in the upper interlayer forms a distorted trigonal prismatic coordination with S atoms, while the intercalated Cr atom in the lower interlayer forms a distorted trigonal antiprismatic coordination with S atoms (Fig. 4l). During the sliding of central CrS_2_ layer, the coordination environment of self-intercalated Cr atoms changes accordingly. The final state is obtained as the upper self-intercalated Cr atoms form a distorted trigonal antiprismatic coordination with S atoms and the lower self-intercalated Cr atoms form a distorted trigonal prismatic coordination with S atoms. The plane-averaged charge densities along *z* direction are plotted in Supplementary Fig. 27b to clarify the charge distribution of two polarization states. For the initial state, the charge near the lower intercalated Cr atom is more than that of the upper intercalated Cr atom, whereas the final state is opposite. The net charge between upper and lower intercalated Cr atoms results in the interfacial charge transfer and induces the spontaneous polarization. The energy difference between polar and non-polar structure is calculated to be 1.4 eV/cell (comparable to that of Bi_6_O_9_ film^46^), implying the relatively high stability of polar phase. In addition, the remanent polarization value of one-unit-cell thick Cr_2_S_3_ is calculated to be 0.10 μC/cm^2^, consistent with the experimental result (Supplementary Fig. 27c).

### Ferromagnetism in 2D non-layered Cr_2_S_3_

**Fig. 5:**
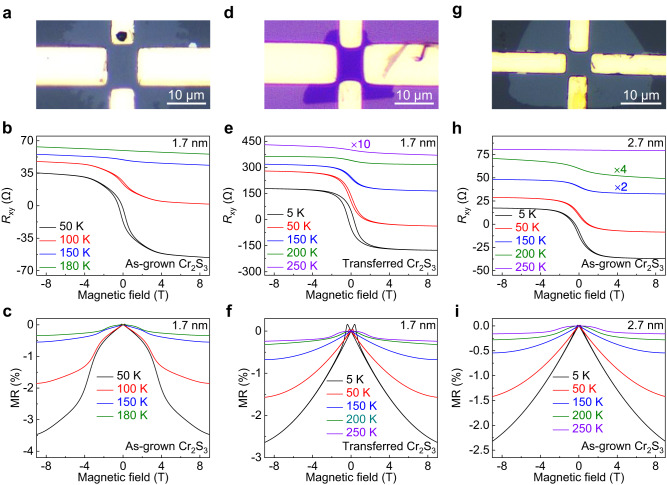
Ferromagnetism in CVD-synthesized one-unit-cell of non-layered Cr_2_S_3_. **a**, **d** OM images of Hall-bar devices for as-grown and transferred one-unit-cell of Cr_2_S_3_ nanosheets. **b**, **e** Anomalous Hall effects of as-grown and transferred one-unit-cell of Cr_2_S_3_ at various temperatures. The hysteresis below critical temperature indicates the ferromagnetism. **c**, **f** MRs of as-grown and transferred one-unit-cell of Cr_2_S_3_ at various temperatures. **g** OM image of Hall-bar device for an as-grown Cr_2_S_3_ nanosheet with the thickness of 2.7 nm. **h**, **i** Corresponding anomalous Hall effect and MR at various temperatures.

2D magnetic materials have shown an unprecedented potential for constructing high-performance non-volatile spintronic memory devices^7,47,48^. Nevertheless, the relatively low Curie temperature and inferior environmental stability fall short of meeting the criteria for practical applications. The abundant phase states and unique self-intercalation of Cr atoms in Cr_*m*_X_*n*_ provide a new paradigm for modulating ferromagnetism. In this regard, low-temperature quantum transport measurements were performed to investigate the magnetism of one-unit-cell thick Cr_2_S_3_ and the OM image of Hall-bar device is displayed in Fig. 5a. As shown in Fig. 5b, as the magnetic field below critical values, the Hall resistances (*R*_xy_) increase with decreasing the magnetic field. And then, when the magnetic fields are above critical values, the anomalous Hall effects evidenced by the saturated Hall resistance plateau are observed up to 150 K, indicating the spontaneous magnetization and long-range ferromagnetic order in one-unit-cell of Cr_2_S_3_. Moreover, the negative magnetoresistances (MRs) are also detected below 150 K, confirming the emergence of ferromagnetism (Fig. 5c).

To eliminate the interfacial effect between Cr_2_S_3_ and sapphire surface, the magneto-transport properties of a transferred Cr_2_S_3_ nanosheet onto SiO_2_/Si were then explored (Fig. 5d). The remarkable anomalous Hall effects and negative MRs are unambiguously detected, which suggests the robust ferromagnetism in Cr_2_S_3_ even down to one-unit-cell thickness (Fig. 5e, f). In addition, the influence of thickness on ferromagnetism was also clarified. After increasing the thickness to 2.7 nm, the Curie temperature increases to 200 K, almost two times higher than that of bulk counterpart^28^, the intralayer-sliding of self-intercalated Cr atoms should result in such an interesting phenomenon (Fig. 5g–i and Supplementary Figs. 28, 29). The comparison of Curie temperature of Cr_2_S_3_ with other Cr_*m*_X_*n*_ is shown in Table 2. Besides, the environmental stability is crucial for exploring ferromagnetism and constructing multifunctional device, especially at one-unit-cell thickness, and the multiscale characterization results indicate the robust stability of CVD-synthesized 2D Cr_2_S_3_, as shown in Supplementary Fig. 30.

**Table 2:** The comparison of Curie temperature of Cr_2_S_3_ with other Cr_*m*_X_*n*_

Material	Substrate	Thickness	Curie temperature	Method	References
CrSe_2_	WSe_2_	10.8 nm	110 K	Reflective magnetic circular dichroism	^25^
		0.7 nm	65 K		
trigonal Cr_5_Te_8_	SiO_2_/Si	6.0 nm	125 K	Reflective magnetic circular dichroism	^26^
monoclinic Cr_5_Te_8_			150 K		
CrTe_2_	SiO_2_/Si	40.0 nm	179 K	Reflective magnetic circular dichroism	^27^
		3.0 nm	189 K		
Cr_2_S_3_	Mica	Bulk	120 K	Vibrating sample magnetometer	^28^
Cr_2_S_3_	*c*-plane sapphire	1.7 nm	150 K	Anomalous Hall effect	This work
		2.7 nm	200 K		

### Magnetoelectric coupling in 2D non-layered Cr_2_S_3_

**Fig. 6:**
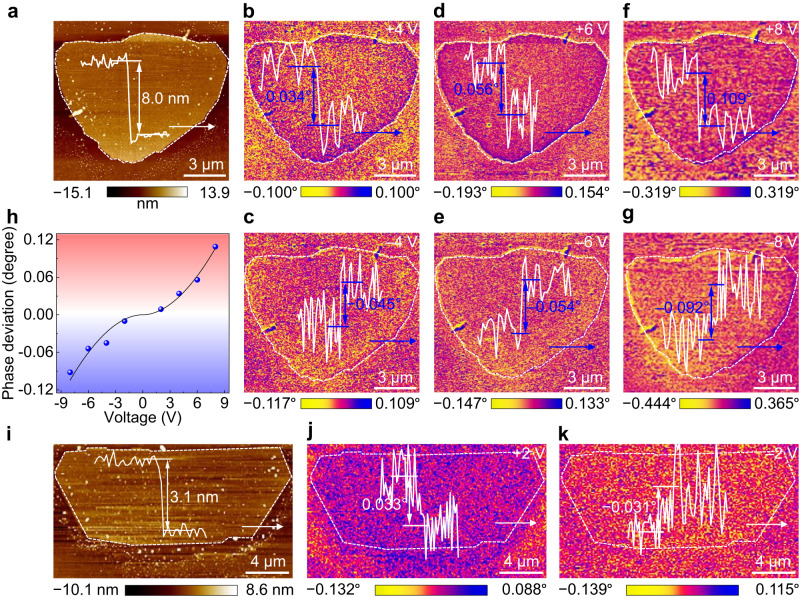
Robust magnetoelectric coupling in 2D non-layered Cr_2_S_3_. **a** AFM image and height profile of a single Cr_2_S_3_ nanosheet with the thickness of 8.0 nm. **b**–**g** MFM phase images of the Cr_2_S_3_ nanosheet, after applying the voltages of ±4 V, ±6 V, and ±8 V, respectively. **h** Voltage-dependent phase deviation of the Cr_2_S_3_ nanosheet. **i** AFM image and height profile of a single Cr_2_S_3_ nanosheet with the thickness of 3.1 nm. **j**, **k** MFM phase images of the Cr_2_S_3_ nanosheet, after applying the voltages of ±2 V, respectively.

Electric field-assisted magnetic force microscopy (MFM) is a nondestructive technology for determining the magnetoelectric coupling in bulk^49^ and 2D multiferroics^50^, and which is performed on as-grown Cr_2_S_3_ with different thicknesses (Fig. 6a, i and Supplementary Fig. 31). After applying a positive voltage (+4 V), the phase deviation between Cr_2_S_3_ and non-magnetic sapphire substrate is detected to be 0.034°, nevertheless, as the voltage changes to –4 V, the phase deviation tunes to be –0.045° (Fig. 6b, c). Further increasing the voltages to ±6 and ±8 V, these phenomena are also observed and large phase deviations are obtained (e.g. 0.056° and –0.054° for +6 and –6 V, 0.109° and –0.092° for +8 and –8 V), as shown in Fig. 6d–g. To further uncover the modulation of electric field on magnetism, the voltage-dependent phase deviations are collected and revealed in Fig. 6h. The variation of phase deviation from positive to negative values is due to the spin flip after applying the electric field with different directions. Several results self-consistently demonstrate the robust magnetoelectric coupling in 2D Cr_2_S_3_ even down to one-unit-cell thick (Fig. 6i–k). The intralayer-sliding of self-intercalated Cr atoms enhances the superexchange interaction of Cr at different positions, which increases the magnetocrystalline energy and Curie temperature of Cr_2_S_3_. Meanwhile, the intralayer-sliding of self-intercalated Cr atoms breaks the space reversal symmetry and induces the *p*-*d* orbital hybridization between S and Cr ions, which result in the emergence of room-temperature ferroelectricity and magnetoelectric coupling in 2D Cr_2_S_3_.

## Discussion

In summary, we design a facile CVD approach to synthesize 1-inch one-unit-cell of non-layered Cr_2_S_3_ with unidirectional orientation on *c*-plane sapphire. The introduction of Cr changes the terminated structure of sapphire surface, increases the interfacial interaction between Cr_2_S_3_ and substrate, induces the parallel steps formation on sapphire surface at low temperature, which contribute to the edge-nucleation of unidirectionally aligned Cr_2_S_3_ nanosheets and single crystal synthesis. Particularly, the interaction between Cr_2_S_3_ and sapphire surface results in the intralayer-sliding of self-intercalated Cr atoms, breaks the space reversal symmetry, and then the room-temperature ferroelectricity emerges and even maintains more than one month. The long-range ferromagnetic order is also obtained with the Curie temperature approaching 200 K. The coexistence and robust coupling of ferroelectricity and ferromagnetism make 1-inch one-unit-cell of non-layered Cr_2_S_3_ the largest and thinnest multiferroic materials. These results present a breakthrough toward the batch synthesis of wafer-scale one-unit-cell of multiferroic single crystals, and open up an avenue for future industrial implementation of multiferroic materials in the next-generation magnetoelectric devices.

## Methods

### Synthesis of unidirectionally aligned Cr_2_S_3_

Before the CVD growth, the *c*-plane sapphire substrates (purchased from Nanjing MKNANO Tech. Co., Ltd.) were cleaned by detergent, deionized water, and ethanol, respectively. The growth of 1-inch one-unit-cell of non-layered Cr_2_S_3_ with unidirectional orientation was conducted in a dual-heating-zone furnace. The sulfur (99.5%, thermo scientific) and Cr powders (99.5%, Innochem) mixed with a few of potassium iodide (KI) particles were selected as the precursors. Notably, the introduction of KI should reduce the evaporating temperature of Cr powders significantly. Before heating, 500 standard cubic centimeters (sccm) argon (Ar) was purged into the chamber for 10 minutes to remove the residual air and humidity. Subsequently, the first and second zones were heated to 170 and 980 °C, respectively, with 110 sccm Ar and 10 sccm hydrogen (H_2_) as the carrier gases. The growth time was set to be 25 min. After completing the CVD growth process, the furnace cover was opened and cooled down to room-temperature.

### Etching-free transfer process

CVD-synthesized one-unit-cell of Cr_2_S_3_ nanosheets were transferred onto target substrates via a PPC-assisted method. In detail, the PPC solution was prepared by dissolving 1 g PPC particles in 5 mL anisole and then was spin-coated onto Cr_2_S_3_/sapphire for 50 s at 2000 rpm/min. After baking the PPC/Cr_2_S_3_/sapphire for 10 min at 90 °C, the edges were scraped for the fast separation of PPC/Cr_2_S_3_ from sapphire surface. The target substrates were used to pick up PPC/Cr_2_S_3_ and then dried at 110 °C. Finally, the PPC/Cr_2_S_3_ was soaked in acetone to remove the PPC supporting layer.

### Multiscale characterizations

The morphology, domain size, thickness, chemical component, and crystalline quality of CVD-synthesized 2D Cr_2_S_3_ were systematically characterized by OM (Olympus BX53M), AFM (Dimension Icon, Bruker), XPS (ESCALAB 250Xi, Mg Kα as the excitation source), SEM (Hitachi S-4800 with the acceleration voltage of 5 KV), Raman spectroscopy (XploRA plus, Horiba with the excitation light of 532 nm), and TEM (JEOL JEM-F200 and JEM-NEOARM with the acceleration voltage of 200 kV). The atomic resolution HAADF-STEM and EDS results were obtained from a spherical-aberration-corrected STEM JEM-ARM200CF with an acceleration voltage of 200 kV. XRD and RSM measurements were performed using a Philips X’ Pert diffractometer.

### Electrical and magneto-transport measurements

The CVD-synthesized Cr_2_S_3_ nanosheets were firstly transferred onto SiO_2_/Si via PPC-assisted method. The devices were fabricated using an ultraviolet maskless lithography machine (TuoTuo Technology (Suzhou) Co., Ltd.). The thermal evaporation system was employed for depositing Cr/Au electrodes with the thicknesses of 5/70 nm. The electrical transport measurements were performed under the vacuum (<1.3 mTorr) and dark conditions using a semiconductor characterization system (Keithley 4200-SCS). Low-temperature quantum transport measurements of Cr_2_S_3_ were conducted in a 9T-Physical Property Measurement System (PPMS, Quantum Design, Dynacool) by constructing a four-terminated Hall bar device. The Hall resistances were measured with the perpendicular magnetic field up to 9 T, and the testing temperature range was set to be 2–250 K with a current of 10 μA.

### Ferroelectric characterizations and magnetoelectric coupling measurements

PFM measurements were carried out under the ambient condition using a constant mode AFM (Bruker Multimode 8) equipped with a Pt/Ir-coated Si cantilever tip (spring constant: 3 N/m). For the local electrical measurements, the bias of ±8 V was applied to the samples. The macroscopic ferroelectric hysteresis loops were measured using the Multiferroic II precision materials analyzer (Radiant Technologies) in a dark box at room-temperature. The electric field-assisted MFM measurements (Dimension Icon with a magnetic CoCr-coated tip in AFM tapping mode, Bruker) were performed on as-grown Cr_2_S_3_ to determine the magnetoelectric coupling with the voltages of 0 ~ ±8 V.

### DFT calculations

All DFT calculations in this work were performed using the Vienna Ab initio Simulation Package (VASP) with projector augmented wave (PAW) potentials^51^. The generalized gradient approximation (GGA) proposed by Perdew, Burke, and Ernzerhof was selected for calculating the exchange-correlation potential^52^. The cut-off energy for plane wave expansion was set to be 520 eV. The energy criterion was set to be 10^−5^ eV in iterative solution of the Kohn-Sham equation. A vacuum layer of 15 Å was added perpendicular to the nanosheet to avoid the artificial interaction between periodic images. The Brillouin zone integration was performed using a 3 × 3 × 1 Monkhorst-Pack *k*-point mesh. All the geometric structures were fully relaxed until the force was below 0.01 eV/Å.

### Ferroelectric calculations

The DFT calculations were performed using the VASP^51^. The ion-electron interactions were interpreted using the PAW approach^53^ and the GGA expressed by the Perdew-Burke-Ernzerhof (PBE) functional was used to describe the exchange-correlation effects^52^. The plane-wave cutoff energy of 500 eV was employed for all the calculations. The convergence criteria were set to be 10^−6^ eV in energy and 0.01 eV/Å in force. The Brillouin zone integration was performed using a 7 × 7 × 1 Monkhorst-Pack *k*-point mesh for one-unit-cell of Cr_2_S_3_. A vacuum layer of more than 15 Å was inserted along the out-of-plane direction to avoid the interactions between periodic images simulated with supercells. The out-of-plane polarization was calculated by the dipole moment correction method. To account for the strong correlation effect involving the localized 3*d* electron, an effective Hubbard parameter (*U*_eff_ = *U* – *J* = 1.5 eV) was applied to Cr atom, where *U* and *J* represented the Coulomb repulsion and exchange parameter, respectively.

### Supplementary information

Supplementary Information

Peer Review File

### Source data

Source Data

## Data Availability

Numerical data underlying the figures presented in this study are provided in the Source data file. All data are available upon request from the corresponding author. Source data are provided with this paper.
