# Supplementary material for: Robust multiferroic in interfacial modulation synthesized wafer-scale one-unit-cell of chromium sulfide
Source: Nat Commun. 2024 Jan 24;15:721. doi: 10.1038/s41467-024-44929-5 (PMC10808545; doi:10.1038/s41467-024-44929-5)
Supplement: Supplementary file 1 — Supplementary Information [file 41467_2024_44929_MOESM1_ESM.pdf]

Supplementary Information

for

Robust multiferroic in interfacial modulation synthesized wafer-scale one-unit-cell of chromium sulfide

Luying Song[1#], Ying Zhao[2#], Bingqian Xu[3], Ruofan Du[1], Hui Li[1], Wang Feng[1], Junbo Yang[1], Xiaohui Li[1], Zijia Liu[3], Xia Wen[1], Yanan Peng[1], Yuzhu Wang[1], Hang Sun[1], Ling Huang[1], Yulin Jiang[1], Yao Cai[4], Xue Jiang[2], Jianping Shi[1]*, Jun He[3]*

[1]The Institute for Advanced Studies, Wuhan University, Wuhan 430072, China.

[2]Key Laboratory of Materials Modification by Laser, Ion and Electron Beams (Ministry of Education), Dalian University of Technology, Dalian 116024, China.

[3]Key Laboratory of Artificial Micro- and Nano-Structures of Ministry of Education, School of Physics and Technology, Wuhan University, Wuhan 430072, China.

[4]The Institute of Technological Sciences, Wuhan University 430072, Wuhan, China

*Address correspondences: jianpingshi@whu.edu.cn, He-jun@whu.edu.cn

[#]These authors contributed equally: Luying Song, Ying Zhao

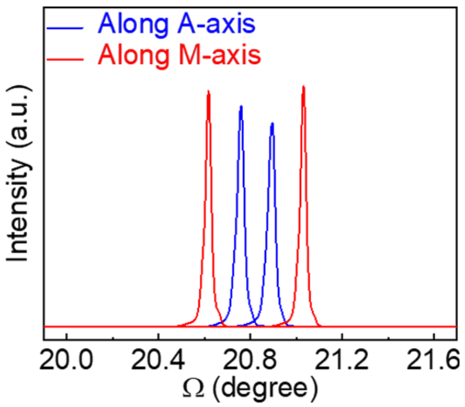

Supplementary Fig. 1 | XRD rocking curves of *c*-plane sapphire. The miscut angles towards *A* and *M* axis are measured to be 0° and 0.2°, respectively.

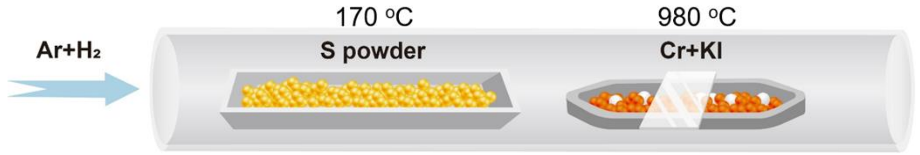

Supplementary Fig. 2 | Schematic diagram of CVD synthesis of 1-inch one-unit-cell of Cr_2S_3 with unidirectional orientation on *c*-plane sapphire. The temperatures of S and Cr powders are set to be 170 and 980 °C, respectively. The KI particles are used to reduce the evaporating temperature of Cr powders.

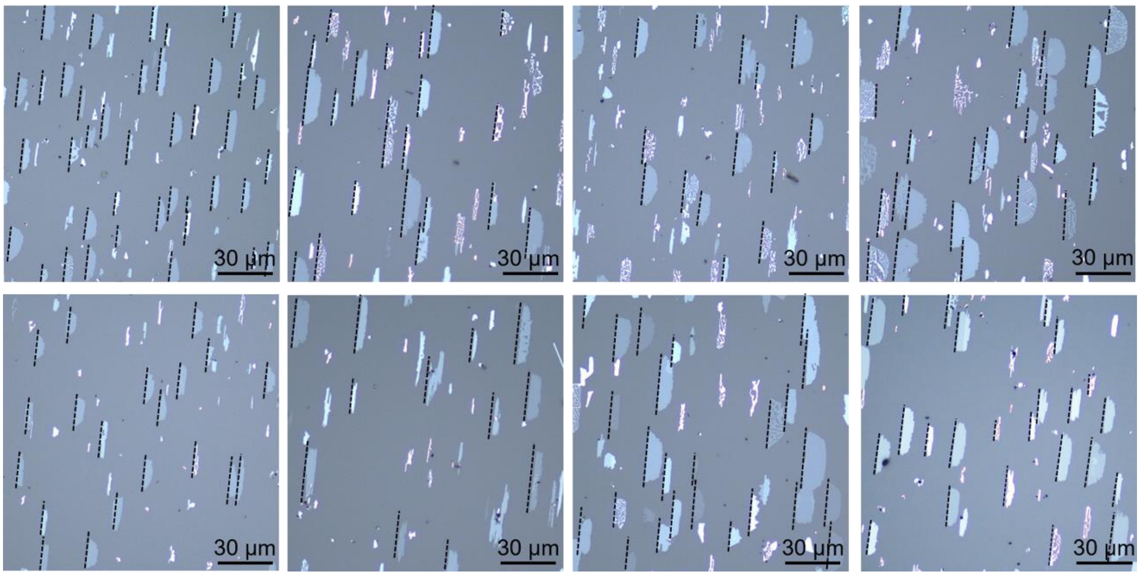

Supplementary Fig. 3 | OM images of as-grown Cr_2S_3 nanosheets captured from different areas of 1-inch *c*-plane sapphire. The unidirectional alignment feature of one-unit-cell of Cr_2S_3 is further confirmed.

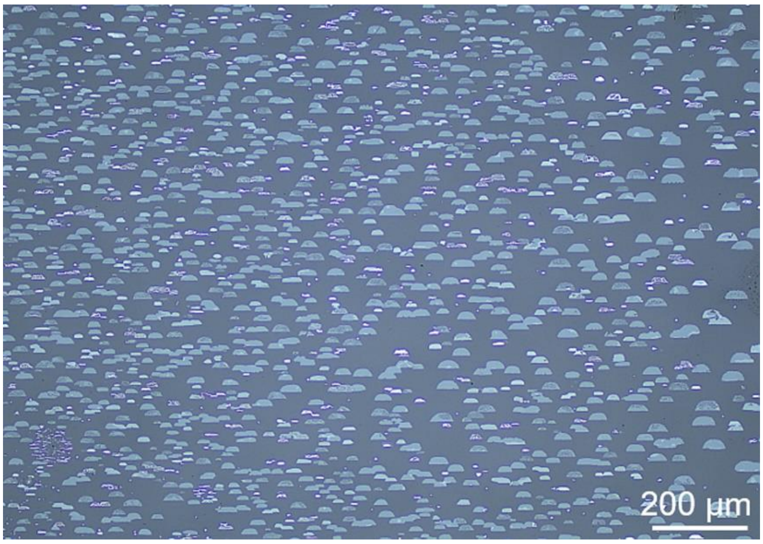

Supplementary Fig. 4 | Large-area OM image of as-grown one-unit-cell of Cr_2S_3 nanosheets with unidirectional alignment on 1-inch *c*-plane sapphire.

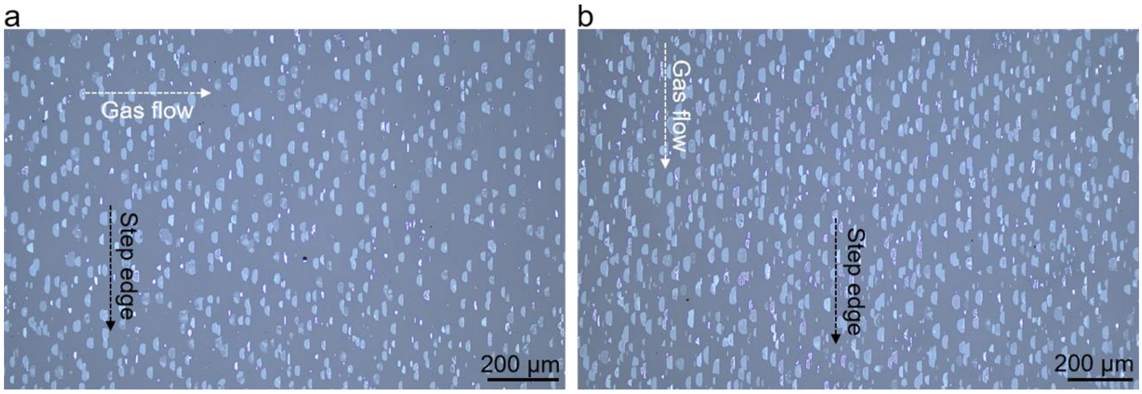

Supplementary Fig. 5 | OM images of as-grown one-unit-cell of Cr_2S_3 nanosheets on *c*-plane sapphire with the carrier gas flow direction vertical (a) and parallel (b) to the step edge direction of sapphire, respectively. The carrier gas flow direction reveals negligible influence on the domain orientation of Cr_2S_3.

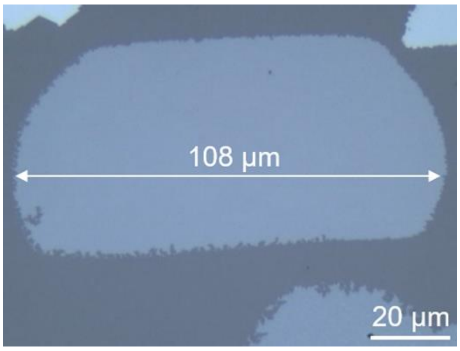

Supplementary Fig. 6 | OM image of an as-grown one-unit-cell of Cr_2S_3 nanosheet on *c*-plane sapphire with the domain size of 108 µm.

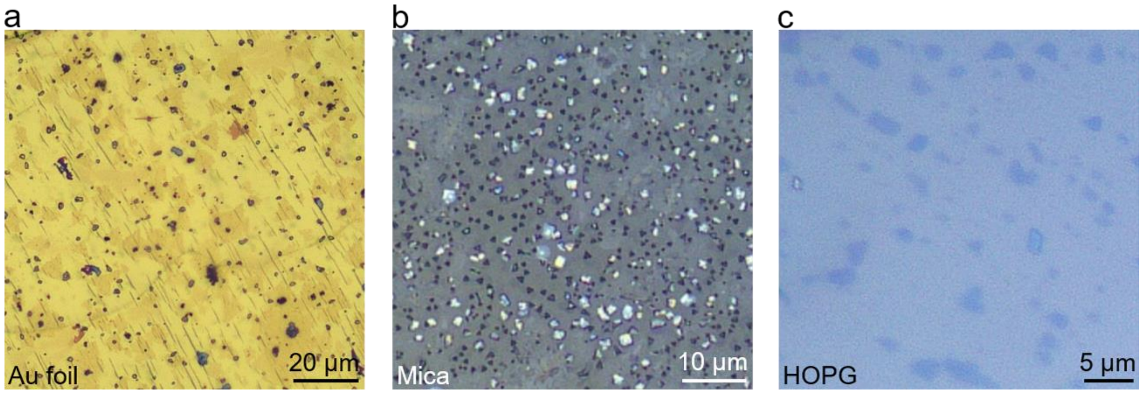

Supplementary Fig. 7 | OM images of as-grown Cr_2S_3 nanosheets on diverse substrates including (a) Au foil, (b) mica, and (c) HOPG. The small domain sized triangular or irregular Cr_2S_3 nanosheets with random orientations are evolved, indicating the advantage of sapphire for the growth of unidirectionally aligned one-unit-cell of Cr_2S_3.

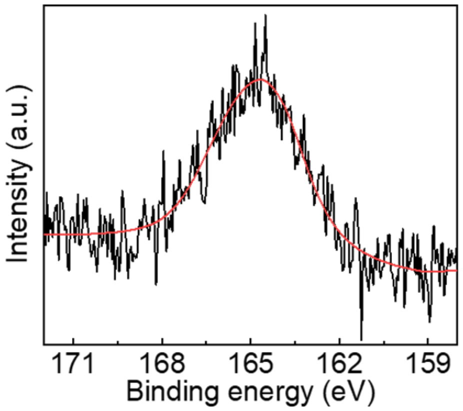

Supplementary Fig. 8 | XPS spectrum of Al_2S_3 powders. The characteristic peak at 164.0 eV is also observed for the as-grown Cr_2S_3 on *c*-plane sapphire (Fig. 1f), indicating the formation of Al-S bonds and the strong interfacial interaction between Cr_2S_3 and sapphire substrate.

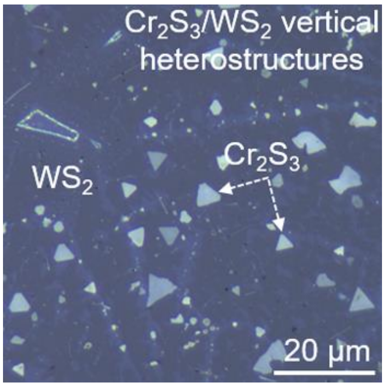

Supplementary Fig. 9 | OM image of as-grown Cr_2S_3/WS_2 vertical heterostructures on *c*-plane sapphire, showing the random orientations of Cr_2S_3 nanosheets.

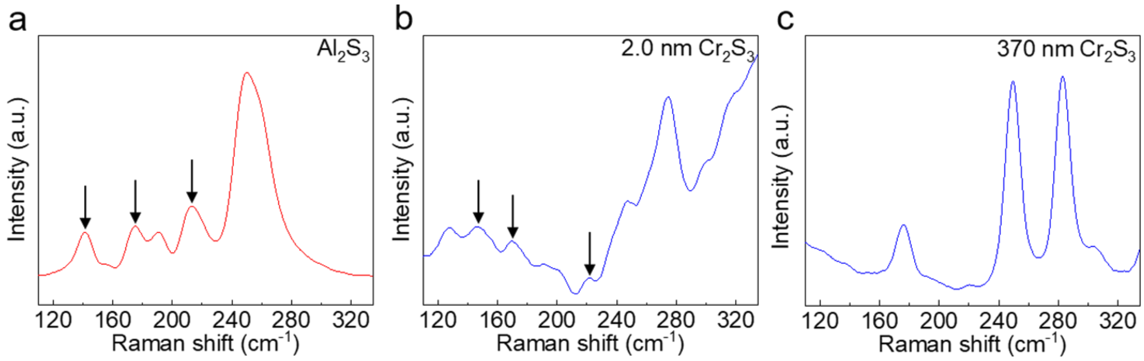

Supplementary Fig. 10 | Raman spectra of Al_2S_3 powders (a), one-unit-cell of Cr_2S_3 (b) and 370.0 nm Cr_2S_3 (c) on *c*-plane sapphire. The nearly identical characteristic peaks in (a) and (b) indicates the formation of Al-S bonds (indicated by the black arrows). For the 370.0 nm Cr_2S_3, these peaks are not observed due to the detection depth limit of Raman spectroscopy.

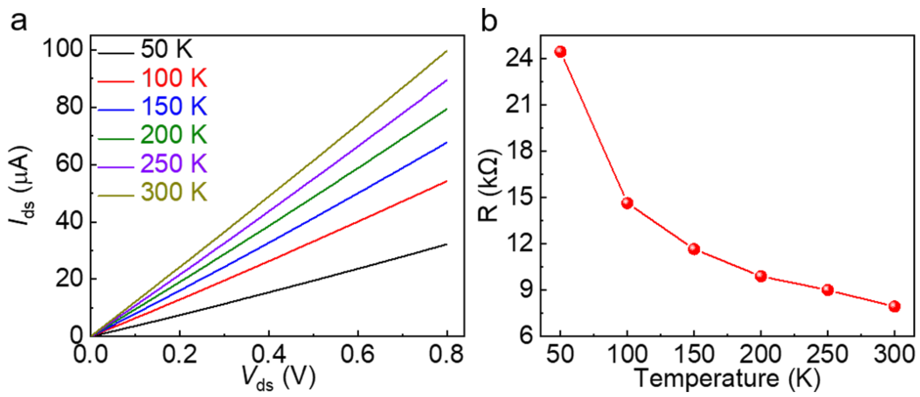

Supplementary Fig. 11 | The semiconducting determination of CVD-synthesized one-unit-cell of Cr_2S_3. a, Temperature-dependent output characteristic curves of a Cr_2S_3 back-gated FET. **b**, Plot of the resistance as a function of temperature.

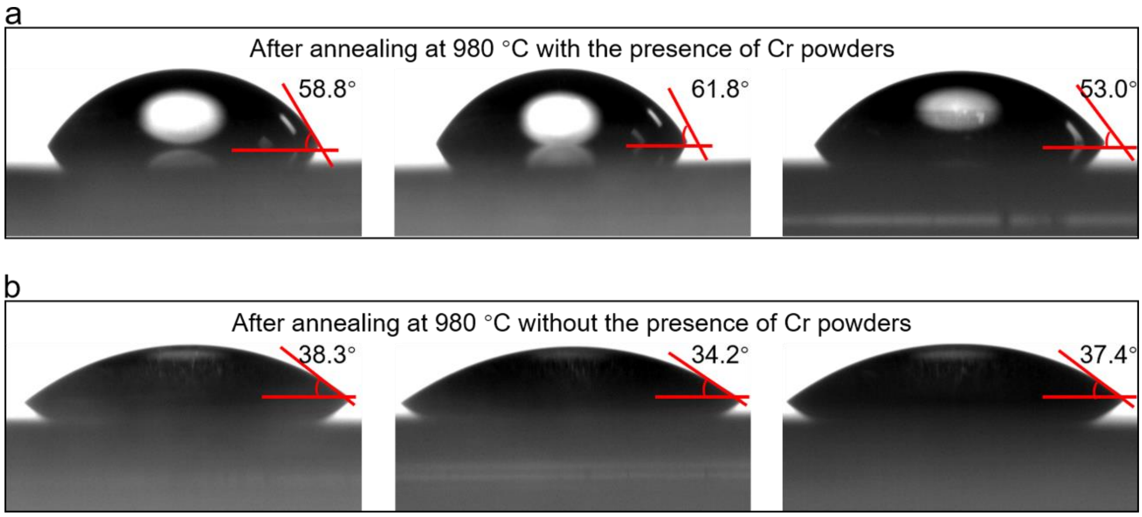

Supplementary Fig. 12 | Water contact angle measurements on two distinct sapphire surfaces. a, Contact angles of distilled water droplet on the sapphire surface after annealing at 980 °C with the presence of Cr powders. **b**, Contact angles of distilled water droplet on the sapphire surface after annealing at 980 °C without the presence of Cr powders.

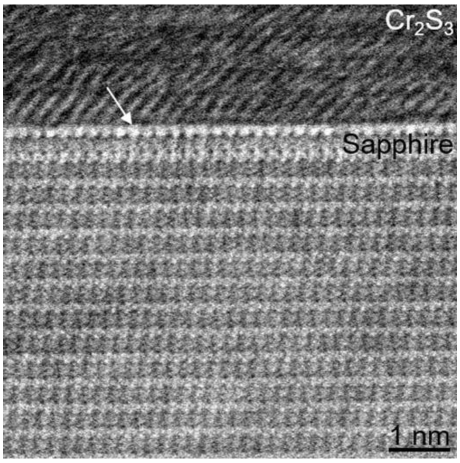

Supplementary Fig. 13 | Atomic-resolution cross-sectional STEM image of as-grown Cr_2S_3 on *c*-plane sapphire. The surface terminated Al atoms are directly observed, as indicated by the white arrow.

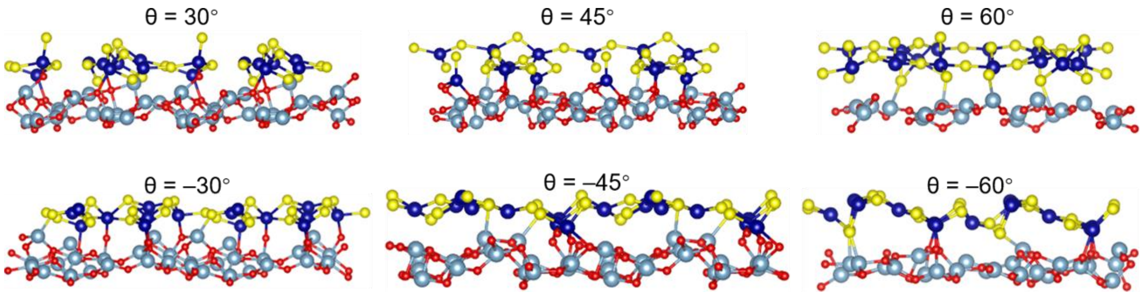

Supplementary Fig. 14 | Atomic structure models of Cr_2S_3 with different rotation angles on Al-terminated sapphire surface. θ is defined as the angle between [110] direction of Cr_2S_3 and [11–20] direction of *c*-plane sapphire.

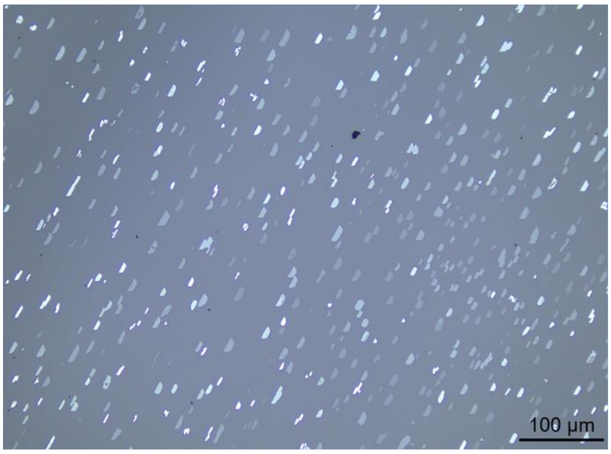

Supplementary Fig. 15 | Large-area OM image of as-grown Cr_2Se_3 nanosheets with unidirectional alignment on *c*-plane sapphire, showing the universality of this interface modulated growth strategy.

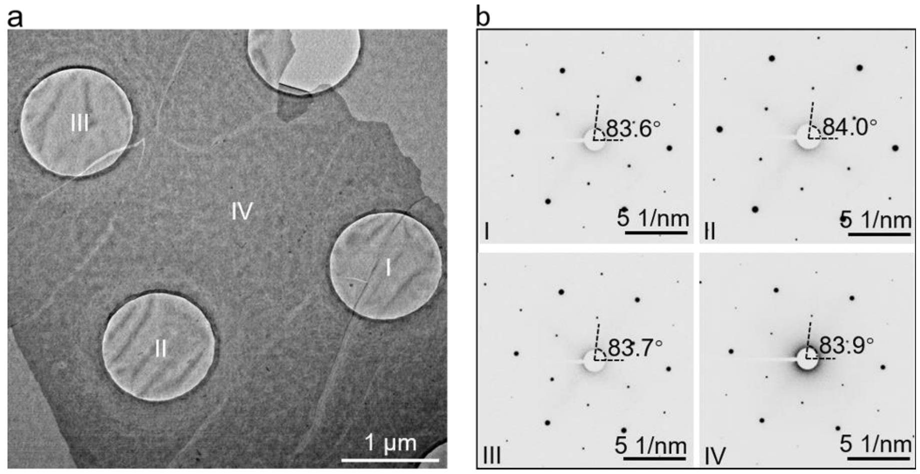

Supplementary Fig. 16 | The single-crystal determination of an isolated Cr_2S_3 nanosheet. a, Low-magnification TEM image of a transferred Cr_2S_3 nanosheet. **b**, SAED patterns captured from different areas of the Cr_2S_3 nanosheet. The close angles within a deviation of 0.4° in the SAED patterns prove that the obtained Cr_2S_3 is single crystal.

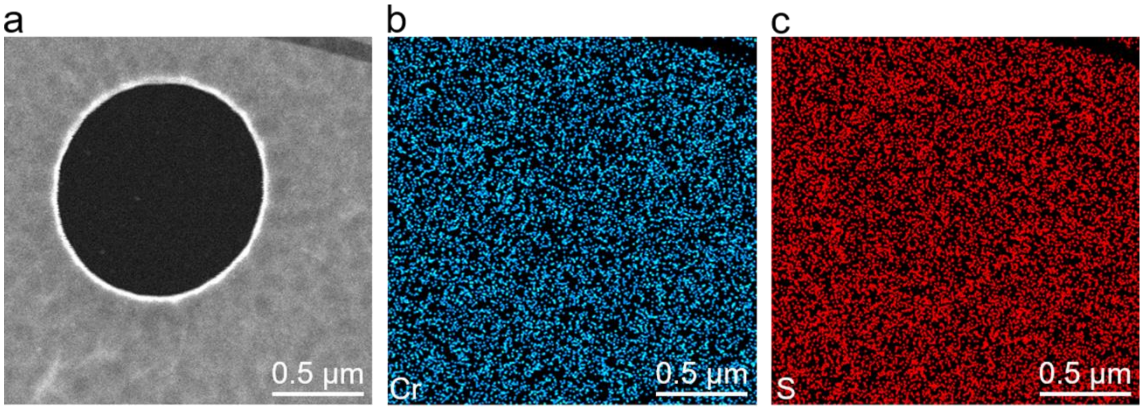

Supplementary Fig. 17 | The element distribution determination of Cr_2S_3. a, Low-magnification STEM image of a transferred Cr_2S_3 nanosheet. **b**,**c**, Corresponding EDS mapping images of Cr and S, showing the uniform element distributions.

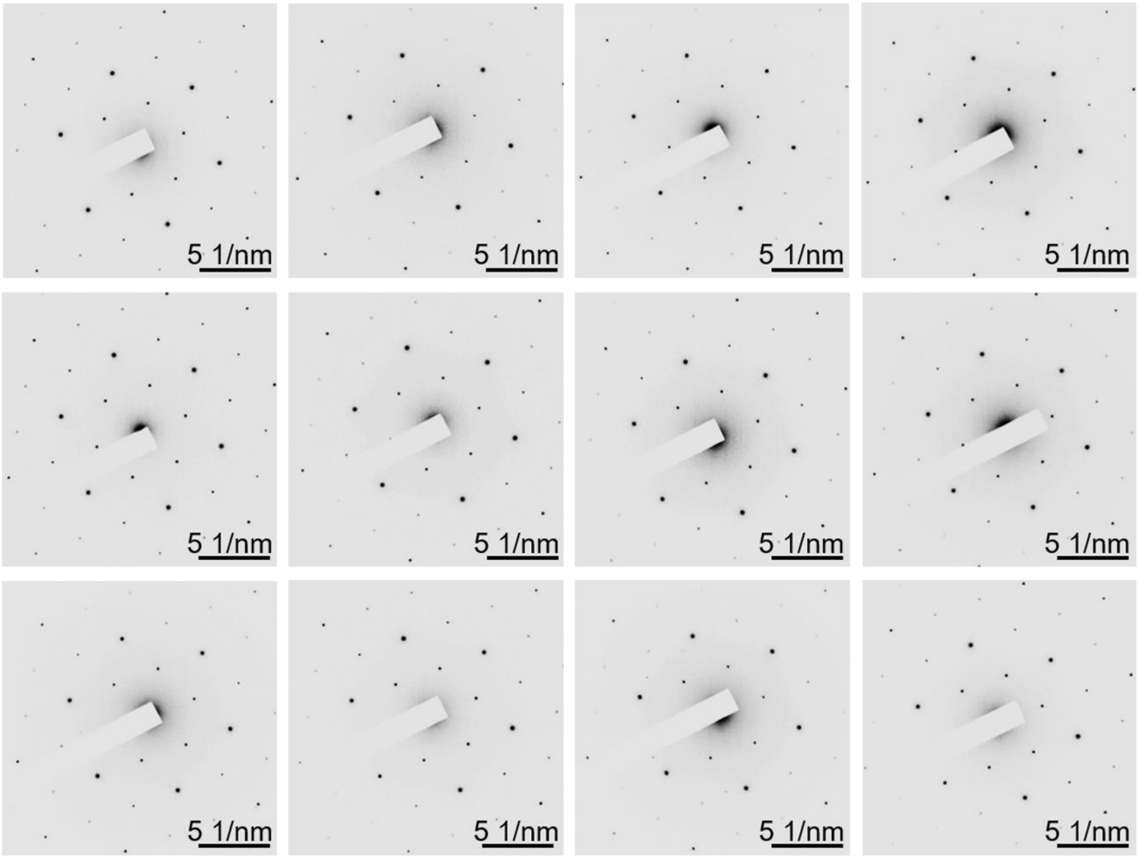

Supplementary Fig. 18 | The determination of unidirectionally aligned Cr_2S_3 nanosheets. A series of SAED patterns captured from different nanosheets show nearly the identical orientations, indicating the unidirectional alignment of Cr_2S_3.

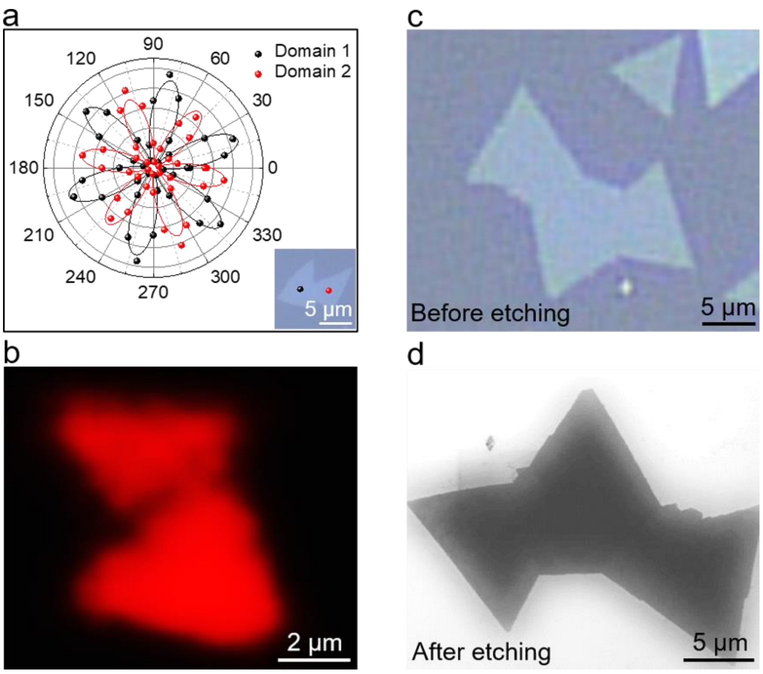

Supplementary Fig. 19 | The defective stitching of Cr_2S_3 nanosheets with random orientations. **a**,**b**, Polarized SHG spectra and mapping image measured on two merged Cr_2S_3 domains with random orientations. Inset: OM image of such two merged domains. **c**,**d**, OM and SEM images of two merged Cr_2S_3 domains with random orientations before and after Ar/O_2 etching.

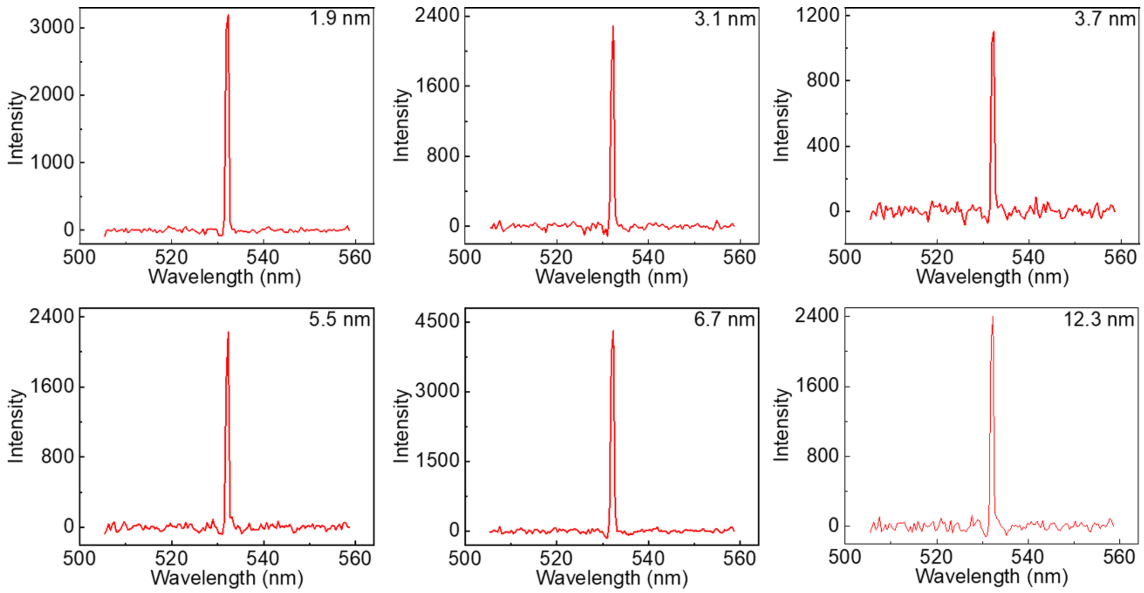

Supplementary Fig. 20 | SHG characterizations of as-grown Cr_2S_3 nanosheets with different thicknesses, showing the non-centrosymmetric structure.

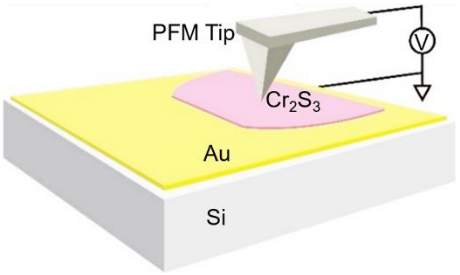

Supplementary Fig. 21 | Schematic diagram of the PFM measurement. The Cr_2S_3 nanosheet is transferred onto Au/Si substrate for the further PFM characterization.

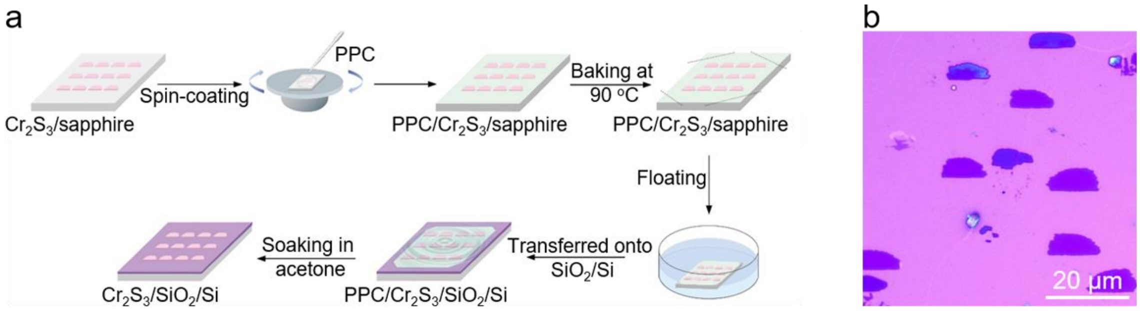

Supplementary Fig. 22 | PPC-assisted etching-free transfer of Cr_2S_3 nanosheets. a, Schematic diagram of the etching-free transfer process. **b**, OM image of transferred Cr_2S_3 nanosheets onto SiO_2/Si substrates.

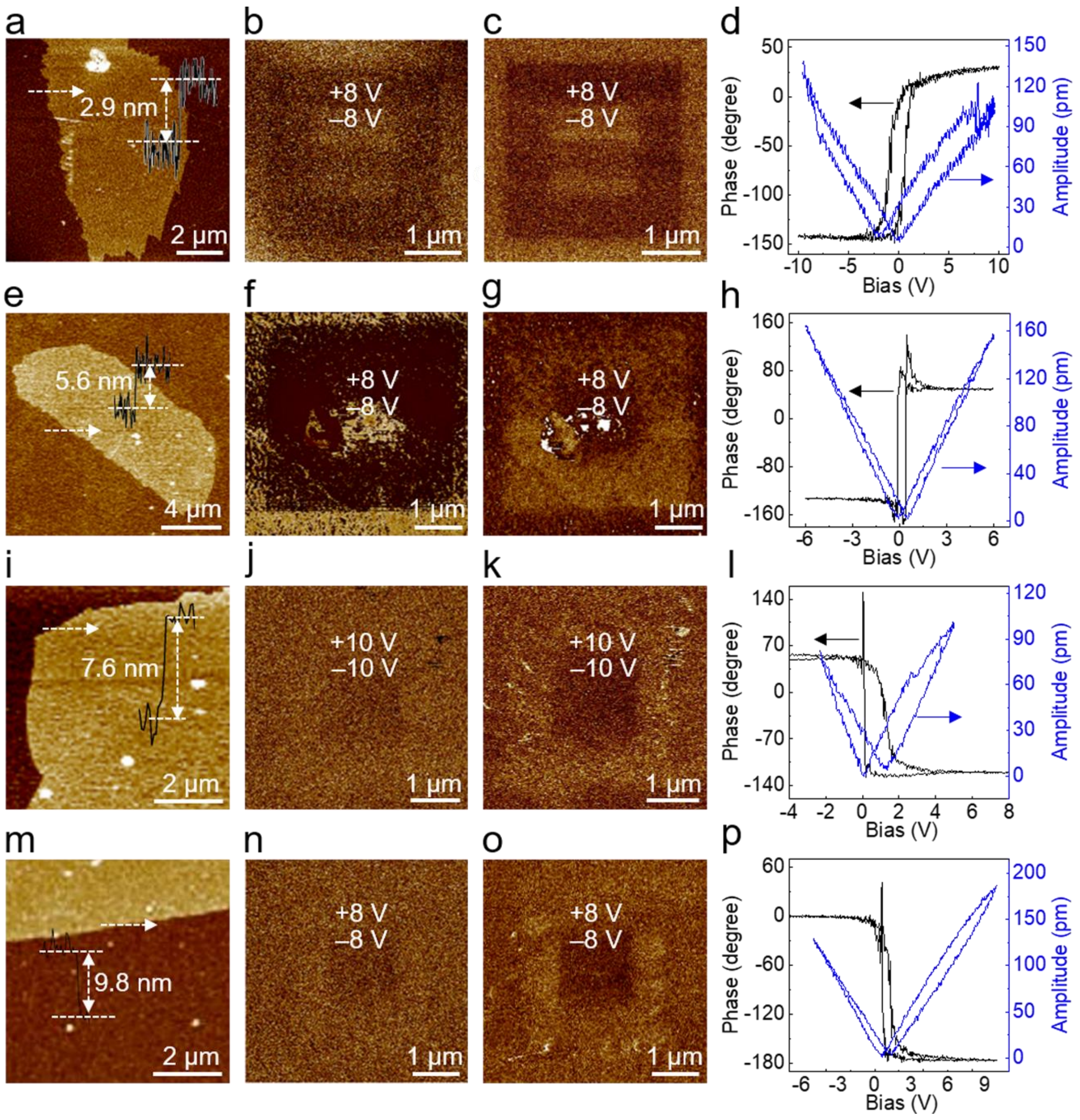

Supplementary Fig. 23 | PFM characterizations of Cr_2S_3 nanosheets with different thicknesses. **a**,**e**,**i**,**m**, AFM images and height profiles of Cr_2S_3 nanosheets with the thicknesses of 2.9, 5.6, 7.6 and 9.8 nm, respectively. **b**,**f**,**j**,**n**, Corresponding PFM phase images. **c**,**g**,**k**,**o**, Corresponding PFM amplitude images. **d**,**h**,**l**,**p**, PFM phase and amplitude hysteresis loops of Cr_2S_3 nanosheets with the thicknesses of 2.9, 5.6, 7.6 and 9.8 nm, respectively.

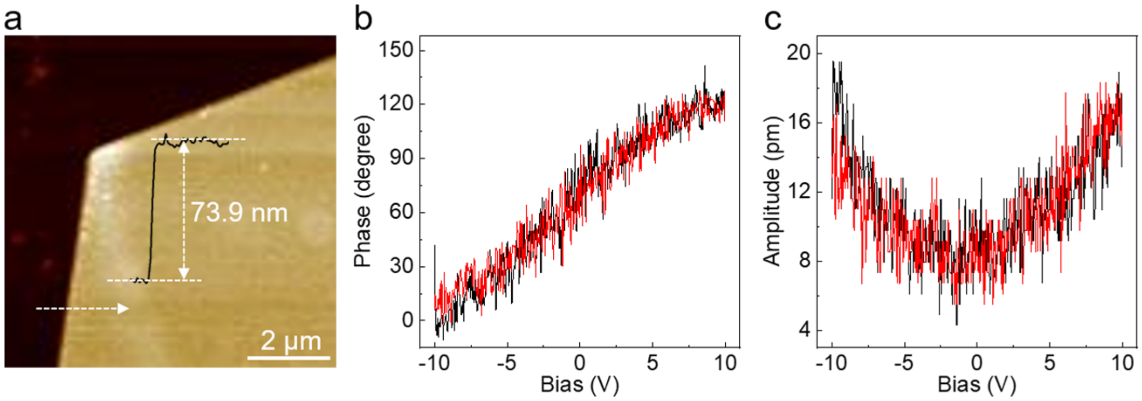

Supplementary Fig. 24 | PFM characterizations of a Cr_2S_3 nanosheet with the thickness of 73.9 nm. a, AFM image and height profile of a Cr_2S_3 nanosheet with the thickness of 73.9 nm. **b**,**c**, Corresponding PFM phase and amplitude hysteresis loops.

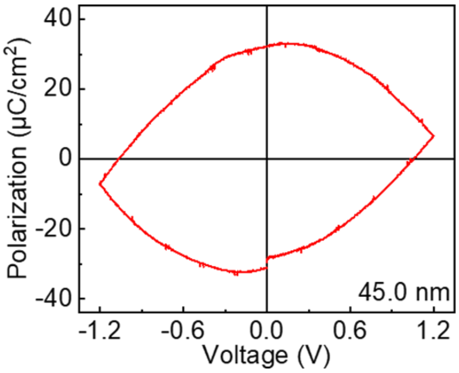

Supplementary Fig. 25 | Macroscopic polarization hysteresis loop of a Cr_2S_3 nanosheet with the thickness of 45.0 nm.

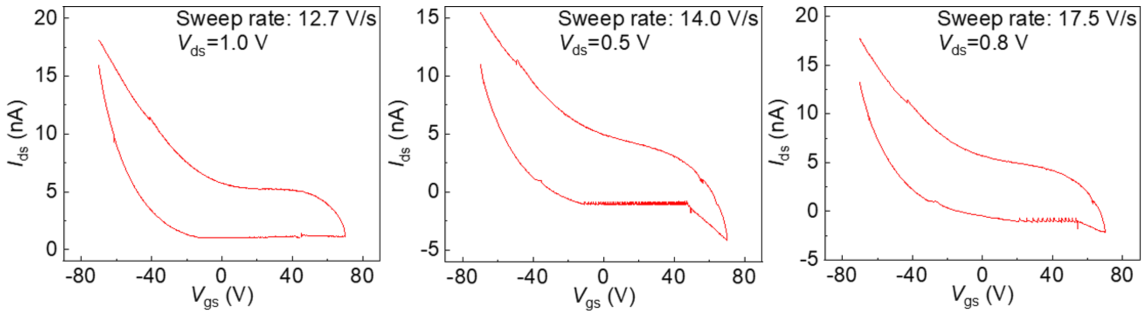

Supplementary Fig. 26 | Transfer characteristic curves of a Cr_2S_3 vertical FET at different sweep rates and drain-source voltages. The counterclockwise hysteresis loops of transfer characteristic curves at different sweep rates and drain-source voltages are observed, and the large hysteresis window (40 V) further verifies the ferroelectric polarization in Cr_2S_3. Besides, the relatively high current on/off ratio (18) indicates the potential applications of Cr_2S_3 in memory devices.

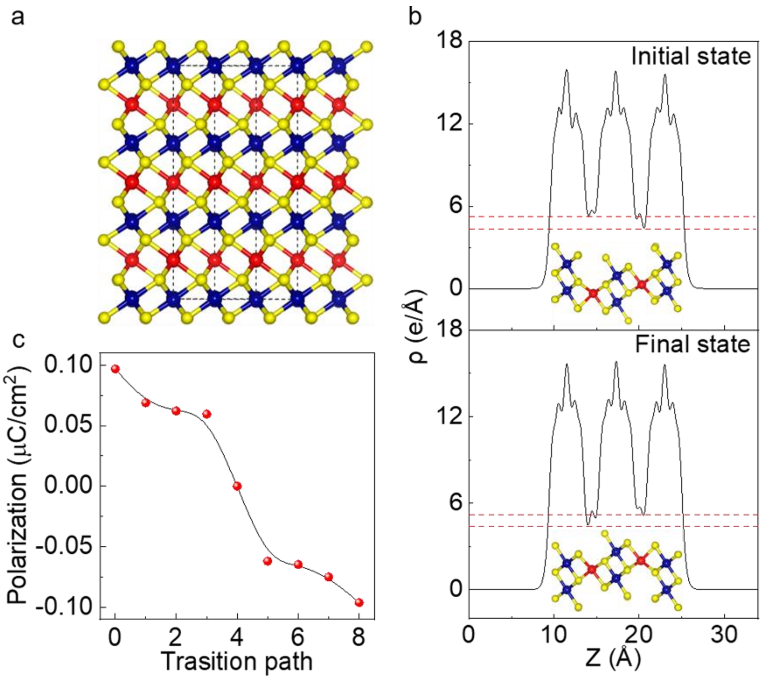

Supplementary Fig. 27 | Polarization reversion mechanism of one-unit-cell of Cr_2S_3. a, Atomic structure of one-unit-cell of Cr_2S_3 without intralayer-sliding of self-intercalated Cr atoms. **b**, Plane-averaged charge densities along *z* direction. **c**, DFT calculated the remanent polarization of one-unit-cell thick Cr_2S_3.

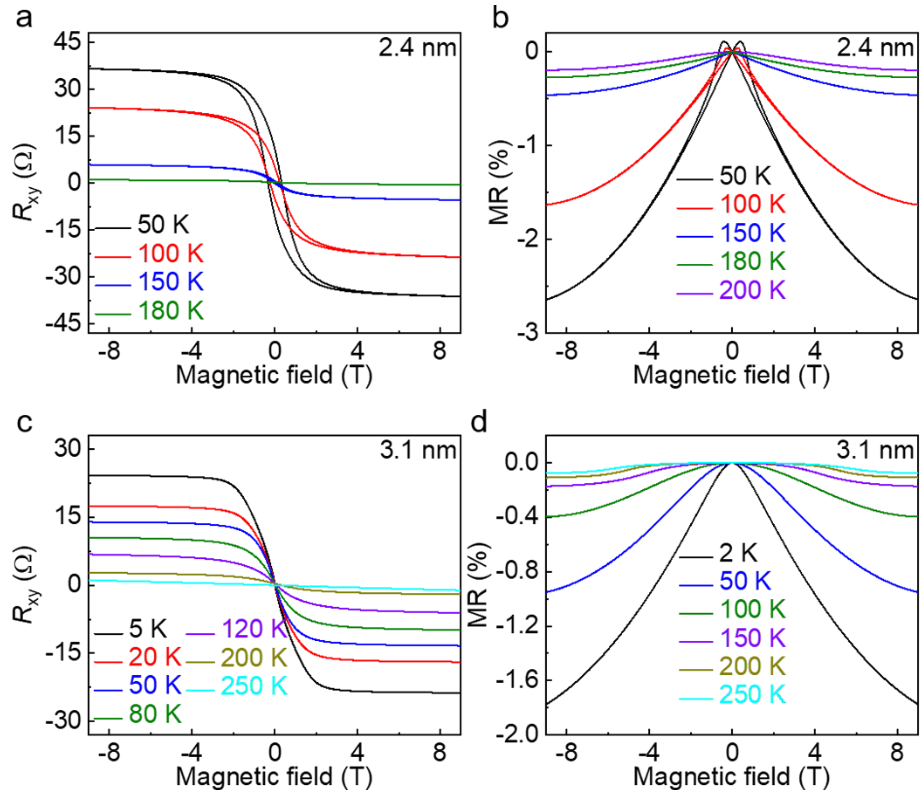

Supplementary Fig. 28 | Robust ferromagnetism in CVD-synthesized Cr_2S_3 nanosheets with different thicknesses. a,**c**, Anomalous Hall effects of as-grown Cr_2S_3 nanosheets with the thicknesses of 2.4 and 3.1 nm, respectively. **b**,**d**, MRs of as-grown Cr_2S_3 nanosheets with the thicknesses of 2.4 and 3.1 nm, respectively.

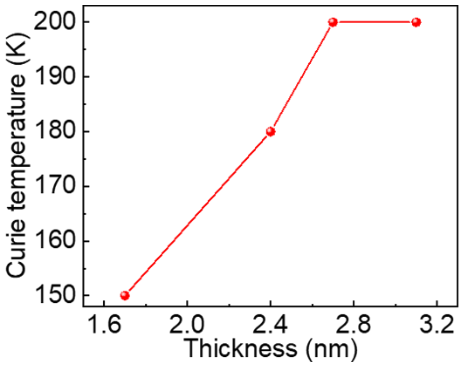

Supplementary Fig. 29 | Phase diagram regarding the Curie temperature of 2D Cr_2S_3 with the thickness.

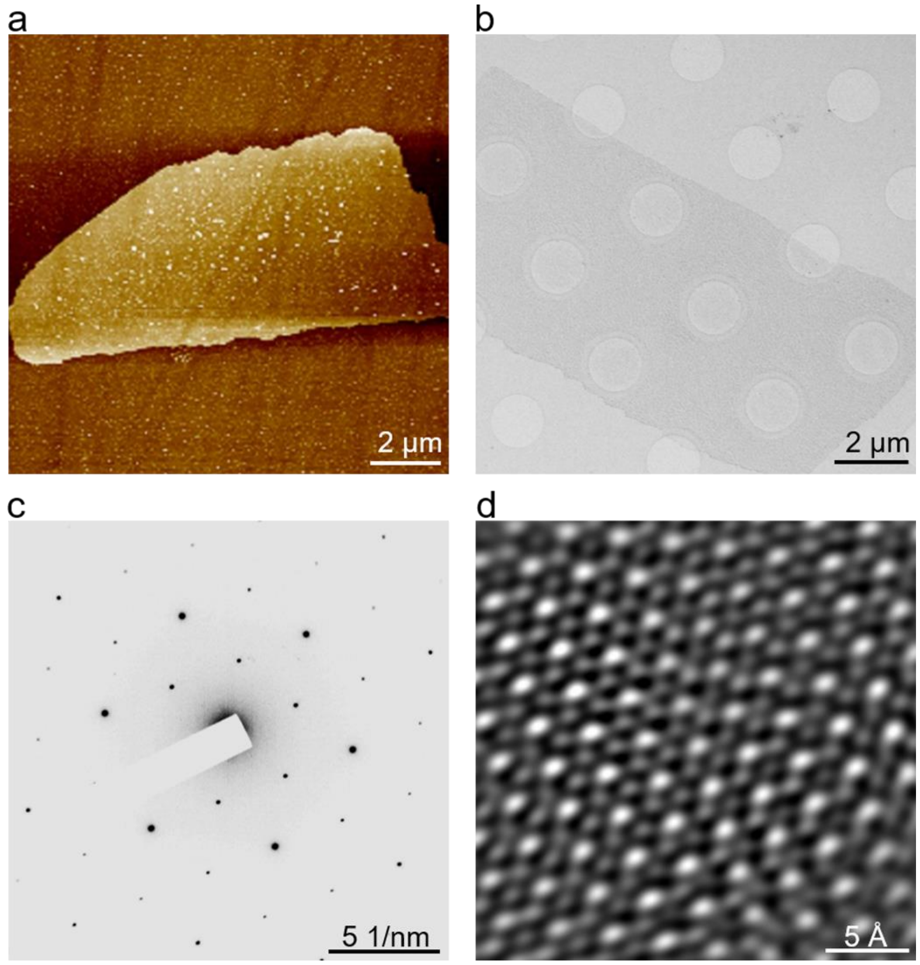

Supplementary Fig. 30 | Environmental stability determination of CVD-synthesized 2D Cr_2S_3. a, AFM image of a seven months aged Cr_2S_3 nanosheet. **b**, Low-magnification TEM image of a seven months aged Cr_2S_3 nanosheet. **c**,**d**, Corresponding SAED pattern and atomic-resolution STEM image, showing its robust environmental stability.

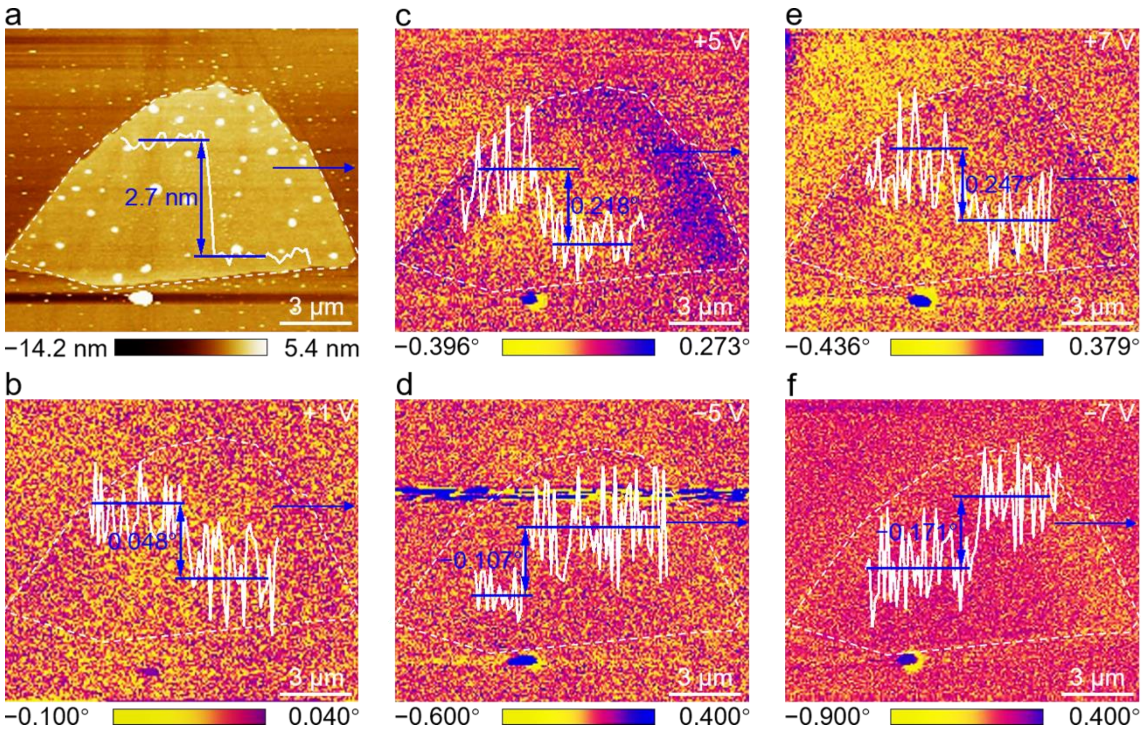

Supplementary Fig. 31 | Magnetoelectric coupling in 2D Cr_2S_3. a, AFM image and height profile of a Cr_2S_3 nanosheet with the thickness of 2.7 nm. **b**, MFM phase image of a Cr_2S_3 nanosheet, after applying the voltage of +1 V. **c**−**f**, MFM phase images of the Cr_2S_3 nanosheet, after applying the voltages of ±5 and ±7 V, respectively.
